# How to use composite indicator and linear programming model for determine sustainable tourism

**DOI:** 10.1186/s40201-017-0271-5

**Published:** 2017-03-28

**Authors:** Maryam Ziaabadi, Mohammad Malakootian, Mohammad Reza Zare Mehrjerdi, Seied Abdolmajid Jalaee, Hosein Mehrabi Boshrabadi

**Affiliations:** 1 Economics of natural resources and Environment, Shahid Bahonar University of Kerman, Kerman, Iran; 2 Environmental Health Engineering Research Center and Department of Environmental Health, School of Public Health, Kerman University of Medical Sciences, Kerman, Iran; 3 Economics of Natural Resources and Environment, Shahid Bahonar University of Kerman, Kerman, Iran; 4 International Economics, Shahid Bahonar University of Kerman, Kerman, Iran; 5 Agricultural Economics-agricultural policy and development, Shahid Bahonar University of Kerman, Kerman, Iran

**Keywords:** Sustainable development, Environmental health, Sustainable indicators, Linear programming model, Tourism

## Abstract

**Background:**

The tourism industry which is one of the most dynamic economic activities in today’s world plays a significant role in the sustainable development. Therefore, in addition to paying attention to tourism, sustainable tourism must be taken into huge account; otherwise, the environment and its health will be damaged irreparably.

**Methods:**

To determine the level of sustainability in this study, indicators of sustainable tourism were first presented in three environmental health, economic and social aspects. Then, the levels of sustainable tourism and environmental sustainability were practically measured in different cities of Kerman Province using a composite indicator, a linear programming model, Delphi method and the questionnaire technique. Finally, the study cities (tourist attractions) were ranked.

**Results:**

Result of this study showed that unfortunately the tourism opportunities were not used appropriately in these cities and tourist destinations, and that environmental aspect (health and environmental sustainability) had very bad situations compared to social and economic aspects. In other words, environmental health had the lowest levels of sustainability.

**Conclusions:**

The environment is a place for all human activities like tourism, social and economic issues; therefore, its stability and health is of great importance. Thus, it is necessary to pay more attention to sustainability of activities, management and environmental health in planning sustainable development in regional and national policy.

## Background

Sustainable tourism is one of the criteria of sustainable development. The tourism industry is considered the biggest and the most diverse industry in the world [1]. Total number of international tourists increased from 25 million in 1950 to 903 million in 2007; the income resulted from this activity reached 865 billion dollars. It is predicted that the total number of tourists will be 1.6 billion people in 2020 [2]. Tourism is a nice experience for visitors and causes employment, income and other benefits for the host community. However, if it is planned or managed inappropriately, it could be a disaster for visitors, the tourism destination and the host community. If the natural or culture environment is damaged or if tourism acts poorly, people lose their positive energy to stabilize and enrich the environment [3, 4]. Sustainable tourism requires systematic attention to environmental, social- cultural and economic aspects so as to use tourist attractions proportional to today’s needs and preservation of future resources [5].

The aim of sustainable tourism is to improve the life quality of host societies, keep equality and justice between two generations and within a generation, maintain the quality of the environment by protecting the ecological system, maintain cultural integrity and social solidarity between communities and create facilities in a way that visitors can have valuable experiences because sustainable development means providing the needs of the existing generation without weakening the ability of future generations to meet their needs [1, 4, 6, 7].

The method of sustainable development is important in tourism planning because tourism is mainly based on attractions and activities that are related to natural environment, historical heritage and patterns of cultural regions. If these resources get harmed or destroyed, tourist resorts cannot attract tourists, and tourism will not be successful [8]. Moreover, poor health conditions decrease economic and social benefits of tourism.

The main objective in the development of sustainable tourism is to provide reasonable methods to utilize natural and human resources and prevent non-scientific use of these resources. The development of sustainable tourism has two aspects of “protection of environment and resources and cultural heritage of societies. Following the Earth Summit in 1992 in Rio de Janeiro that asked the governments to minimize the loss and damage to the environment, they reached an agreement on the agenda of the meeting 21 (plans for 21th century). In fact, the agenda contained a set of detailed action plans that determined each country's role in achieving sustainable development. Therefore based on agenda 21, international organizations of tourism "Agenda 21 for Tourism" was released; it reminded the need to recognize the role of tourism in the appropriate development process and the necessity for practical plans for tourism organizations in order to activate the principles of sustainable tourism towards sustainable development [7, 9–11]. Following this conference, the World Tourism Organization (WTO) started some activities and defined development of sustainable tourism as “sustainable tourism development fulfills the needs of present tourists and host regions and maintains the opportunities of future generations.” Sustainable tourism development manages all resources in a way that it fulfills economic, social and aesthetic needs and maintains cultural interactions, ecological processes and biodiversity and supports systems of environment [12–15]. Perez et al. in Cuban examined sustainable tourism in towards sustainable development [16]. Blancas et al. [9] and Lozano- Oyola et al. [17] in Spain, also, Blancas et al. [18] in coastal areas of Andalusian (Spain) determined level of sustainable tourism using sustainability indicators. Durovic and Loverentjev presented sustainable tourism indicators generally [19]. Xu and Fox in China and UK checked tourism and sustainable development [20]. It is necessary to achieve sustainable tourism by identifying indicators of sustainable tourism in its current status and analyzing the tourism situation and its consequences and effects on the environmental health, society and economy scientifically and carefully [9, 16]. Concerning the above mentioned issues, the sustainability or non-sustainability of tourism elements was examined according to principles and criteria for sustainable tourism and by adopting appropriate policies for it.

**Fig. 1:**
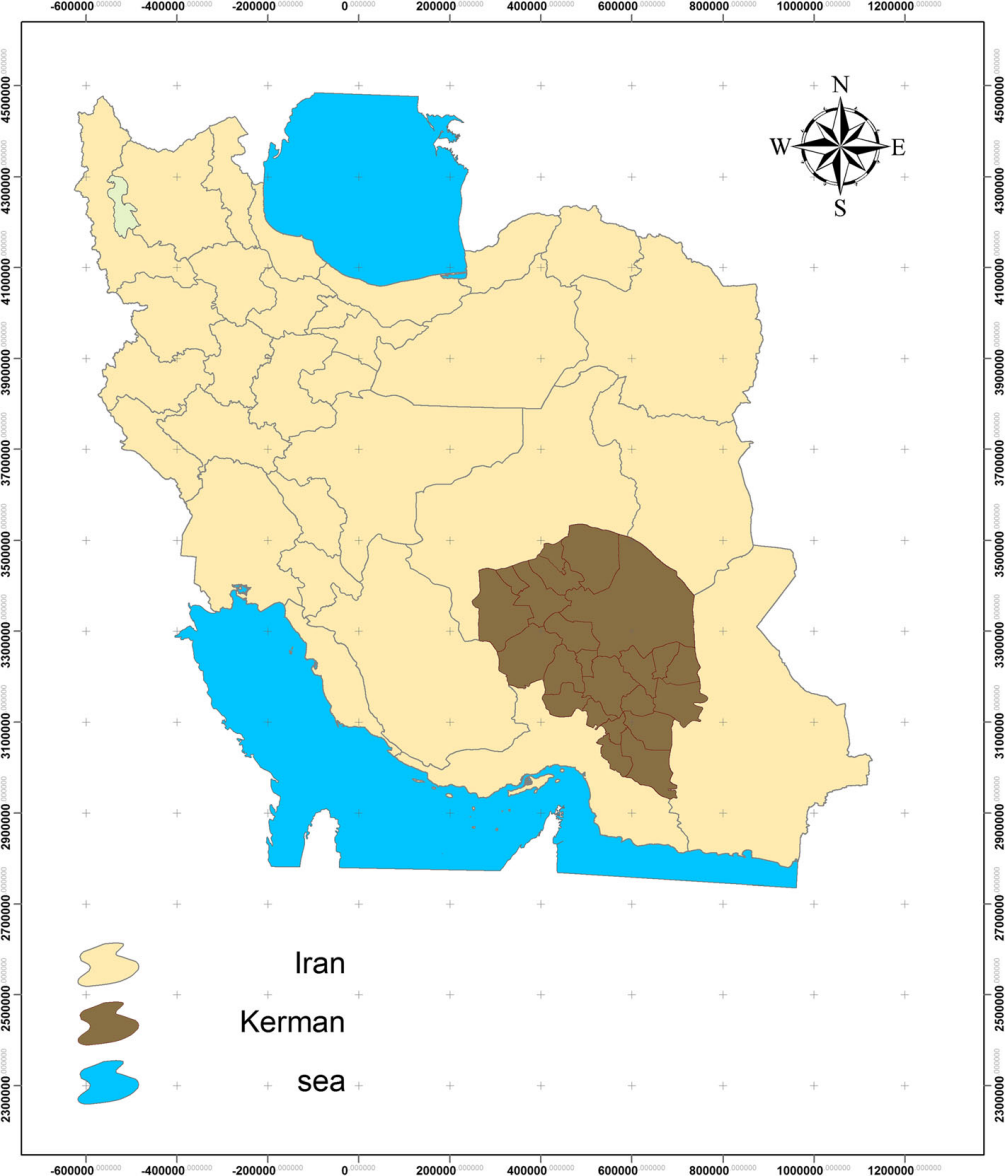
Geographical position of study area

The first purpose of this study was to achieve the appropriate set of indicators for sustainable tourism (especially considering the importance of the environmental health and sustainable development). Moreover, the second goal of this study was to make composite indicator of assessment of sustainable tourism in selected tourist cities of Kerman Province (Fig. 1). This way increased the users' ability to analyze and interpret it practically.

The innovative aspect of this study was to determine the combined indicator in social, economic and environmental aspects; in addition to providing a stable compound index, this indicator was analyzed using linear programming models. Using the Delphi method, which is dependent on field studies, was another aspect of innovation in this research.

## Methods

This cross-sectional study was conducted in 2015 at selected tourist cities of Kerman Province (Kerman, Bam, Jiroft, Rafsanjan, Sirjan and Zarand); these cities were selected due to their historical context, their abundance of natural resources and their more touristic areas compared to other cities. In addition, based on the criteria presented by WTO and other experts, evaluation criteria for operating indicators were 1- being relevant to the subject, 2- data access (collecting and processing capacity) 3-information validity, 4- clearness and understandability for users, and 5- the comparability over time and across jurisdictions or regions [9, 15, 18, 21]. The quantitative data collected through documentary-library methods and statistical yearbooks of Kerman in 2013 required qualitative information by Delphi method and a questionnaire. To design a questionnaire, a large number of sustainable tourism indicators were collected in environmental health, environmental sustainability and social and economic issues by reviewing previous studies. Then, these indicators were localized by experts and professionals opinions and Delphi method through 90 university professors, managers, researchers in the field of tourism and environmental health in cities of Kerman province in three environmental health, social and economic domains (Tables 1, 2, 3) and were scored from 0 to 10 (0, the minimum and 10, the maximum score and significance, for qualitative indicators) [16]. Validity was determined by experts, and stability was analyzed using Cronbach's alpha coefficient [22]. In the first stage (after completing the questionnaires and data collection), composite indicator of sustainable tourism was measured.

**Table 1:** Environmental and environmental health indicators of sustainable tourism

Main factor		Indicator	*I*_*ij*_ (sign)
Environmental health	Access to clean water	The percent of the local people who have access to clean and healthy water	I_i1_(+)
	Water quality of tourism regions	Quality evaluation of water of tourism regions (water pollution) (0–10)	I_i2_(+)
	Waste produced by the tourism sector	Waste per capita (daily)	I_i3_(−)
	Noise pollution	Noise pollution	I_i4_(−)
	Producing of greenhouse gases (air pollution)	Annual emissions of greenhouse gases per capita	I_i5_(+)
	Environmental health (other factors)	Tourists evaluation of environmental health (0–10)	I_i6_(+)
	Environmental damages	People imagination from tourism environmental damages (0–10)	I_i7_(−)
protected natural regions		The percentage of protected natural regions/total natural regions	I_i8_(−)
Ecological assessment of natural resources		Biodiversity and species diversity of flora and fauna (per unit area)	I_i9_(−)
Energy consumption in tourism sector		Energy consumption daily (per capita)	I_i10_(−)
Water consumption in tourism sector		Water consumption daily (per capita)	I_i11_(−)
Construction in region		Construction density in area unit	I_i12_(+)
Erosion		The rate of region erosion	I_i13_(+)
Natural landscapes		The percentage of the region's natural landscapes	I_i14_(+)
Diversity of natural attractions		The number of natural attractions to region area	I_i15_(−)
Agricultural level of region		The percentage of agricultural land to total region area	I_i16_(−)
Intensity of tourism use		The number of tourists in region unit area	I_i17_(−)
Disserted villages of region		Disserted villages/total number of villages	I_i18_(+)
Intensity of natural resources use		The number of tourists in protected region unit area	I_i19_(+)
Planning and environmental management		An environmental administrative unit	(+) I_i20_
environmental awareness level		Assessment of promoting environmental awareness (0–10)	I_i21_(−)
Protection of natural resources and destinationcultural heritage		Budget of cultural heritage-historical (per capita)	I_i22_(+)
Vegetation		Percentage of the region vegetation	I_i23_(+)
Rare plant species		The number of rare plant species (per unit area)	I_i24_(+)
Vegetation		The diversity of plant species (per unit area)	I_i25_(+)
Rare animal species		Rare animal species (per unit area)	I_i26_(+)
Diversity of animal species		The diversity of animal species (per unit area)	I_i27_(+)

**Table 2:** Social indicators of sustainable tourism

Main factor	Indicator	*I*_*ij*_ (sign)
Sport services	Sport gym per capita	(+) I_*i*28_
Health and hygiene services	Hospitals and clinic per capita	(+) I_*i*29_
Transportation services	Transportation vehicle per capita	(+) I_*i*30_
Financial services	Bank per capita	(+) I_*i*31_
Pharmaceutical Services	Pharmacy per capita	(+) I_*i*32_
Tourism benefits	Distribution of tourism benefits for locals (0–10)	(+) I_*i*33_
Tourism benefits	Distribution of tourism benefits for tourists (0–10)	(+) I_*i*34_
Tourism benefits	Distribution of tourism benefits for environment (0–10)	(+) I_*i*35_
Tourism attention to	Number of agencies and tour centers in area (per capita)	(+) I_*i*36_
Participation and cooperation of people for tourism activities	People motivation for participation and cooperation with local tourism organization (0–10)	(+) I_*i*37_
Participation and cooperation of non-governmental organization for tourism activities	The motivation of non-governmental organization for participation in local tourism activities (0–10)	(+) I_*i*38_
Management of tourism activities	Assessment Management of cultural tourism activities in the region (0–10)	(+) I_*i*39_
Management of tourism activities	Assessment Management of ecotourism activities in the region (0–10)	(+) I_*i*40_
Management of tourism activities	Assessment Management of agricultural tourism activities in the region (0–10)	(+) I_*i*41_
Management of tourism activities	Tourism activities share in different economic sectors	I_*i*42_(+)
Tourism share in destination economy	Safety assessment of destination by tourists (0–10)	(+) I_*i*43_
Tourists satisfaction from region safety	The per capita of region safety equipment (ambulance, road emergency)	I_*i*44_(+)
Tourists satisfaction from region safety	Assessment the tension rate between tourists and residents (0–10)	I_*i*45_(‐)
Tourists satisfaction from region safety	The number of recorded crimes in the region (per capita)	I_*i*46_(‐)
The role of t law enforcement in providing security for tourists	Evaluation of military cooperation and local or governmental law enforcement agencies to provide security for tourists (0–10)	I_*i*47_(+)
National and regional advertising	Awareness and positive publicity in the tourism region (0–10)	I_*i*48_(+)
National and regional advertising	Negative publicity for southern regions of the country (southern cities of province) (0–10)	I_*i*49_(‐)
Protecting cultural heritage	Budget of region cultural heritage (per capita)	I_*i*50_(+)
The rate of using cultural heritage	The number of tourists to Antiquities area and cultural heritage	I_*i*51_(‐)
Holding cultural festivals to keep and introduce customs	The number of cultural exhibitions (per capita)	(+) I_*i*52_
Attention to sustainable tourism	Increasing attention of agencies to sustainable tourism (balanced) (0–10)	I_*i*53_(+)
Attention to sustainable tourism	Increasing attention level of policy makers to sustainable tourism (0–10)	I_*i*54_(+)
Attention to sustainable tourism	Tourists motivation for sustainable tourism (0–10)	I_*i*55_(+)
Attention to sustainable tourism	Innovation for sustainable tourism (0–10)	I_*i*56_(+)
Attention to sustainable tourism	Changing attitudes toward environment and the importance of protecting attractions (0–10)	I_*i*57_(+)
Stability of population level	Instability level of the region's population	I_*i*58_(‐)
Young population	The percentage of young population of the region	I_*i*59_(+)
old population	The percentage of old population of the region	I_*i*60_(‐)
The population density	The number of people per unit area	I_*i*61_(‐)
Stability of population level	The net rate of region migration	I_*i*62_(‐)
Stability of population level	Natural rate of population increase	I_*i*63_(‐)
The imposition of a foreign culture	The percentage of foreign population like Afghans in the region	I_*i*64_(‐)
Social tolerance capacity	tourists rate to the region's population (host community)	I_*i*65_(‐)
The impact of social conditions on longevity population	Life expectancy	I_*i*66_(+)
The region income level	The income per capita	I_*i*67_(+)
The family percentage using social utilities in region	The family percentage using social utilities in region (electricity)	I_*i*68_(+)
Understanding and cooperation	Mutual understanding and cooperation of local people with tourists (0–10)	I_*i*69_(+)
Elderly care	Assessment of elderly care facilities in tourism regions (0–10)	I_*i*70_(+)
Children care	Assessment of children care facilities in tourism regions (0–10)	I_*i*71_(+)
Unemployment rate	Unemployment rate of region	I_*i*72_(‐)
Tourism impact on residents	Local people imagination from services improvement because of tourism (0–10)	I_*i*73_(+)
Tourism impact on residents	Local people imagination from the adverse effects of tourism on local people's lifestyle (0–10)	I_*i*74_(‐)
Tourism impact on residents	Local people imagination from tourism impact on avoiding local people exit from region (0–10)	I_*i*75_(+)
Tourism impact on residents	Local people imagination from life quality improvement because of tourism increase in region (0–10)	I_*i*76_(+)
Tourists impression of quality of public services	Tourists impression of quality of public services (accommodation and transport facilities) (0–10)	I_*i*77_(+)
Hospitality and willingness to receive tourists	Hospitality assessment and willingness to receive tourists in the local community (0–10)	I_*i*78_(+)
Conservation, reconstruction and restoration of monuments and cultural heritage	Conservation budget, reconstruction and restoration of monuments and cultural heritage (per capita) (0–10)	I_*i*79_(+)
Environment improvement	Green space per capita	I_*i*80_(+)
Women's rights	Evaluation of improving women's rights (0–10)	I_*i*81_(+)
Labor rights and social security	Evaluation of improving labor rights and social security (0–10)	I_*i*82_(+)
Education level	Universities and higher education centers	I_*i*83_(+)
Education level	Population literacy rate	I_*i*84_(+)
Variety of handicrafts	Evaluation of the number and variety of handicrafts to attract tourists (0–10)	I_*i*85_(+)
Cultural-historic background	The number of cultural-historic sites (per unit area)	I_*i*86_(+)
Cultural-historic background	Local-traditional cultures	I_*i*87_(+)

**Table 3:** Economic indicators for sustainable tourism

Main factor	Indicator	*I*_*ij*_ (sign)
Tourism demand of region	Number of tourist	I_*i*88_(+)
Tourist length of stay	Average of length of stay	I_*i*89_(+)
Tourism income	The expense of one night stand of tourists	I_*i*90_(+)
Income distribution	ini coefficient	I_*i*91_(+)
Tourism satisfaction	The satisfaction of domestic tourists from the region (0–10)	I_*i*92_(+)
Tourism satisfaction	The satisfaction of foreigner tourists from the region (0–10)	I_*i*93_(+)
Tourism satisfaction	Positive imagination of tourists from the relationship between quality and services price in the region (0–10)	I_*i*94_(+)
Tourism satisfaction	The imagination of tourists from the relationship between quality and accommodation price (0–10)	I_*i*95_(+)
Tourism satisfaction	The imagination of tourists from the relationship between quality and restaurant price (0–10)	I_*i*96_(+)
Tourism satisfaction	Assessment work quality of staff in the tourism sector (hotels, restaurants, etc.) (0–10)	I_*i*97_(+)
Tourism satisfaction	Tourist's satisfaction from protected collections and regional cultural collection (0–10)	I_*i*98_(+)
Tourism information transparency	Tourists assessment of transparency of tourism information (0–10)	I_*i*99_(+)
Currency rate	he percentage of currency rate changes (foreigner tourists)	I_*i*100_(+)
Communication conditions	Telecommunication and post facilities (per capita)	I_*i*101_(+)
Communication conditions	Online communication (ADSL) (per capita)	I_*i*102_(+)
Tourism planning	Evaluation of tourism planning in region (0–10)	I_*i*103_(+)
Government participation with non-governmental organization	Evaluation of government participation with non-governmental organization about local tourism activities (0–10)	I_*i*104_(+)
People participation rate and local organization	Evaluation of people participation rate and local organization for providing and executing tourism plans (0–10)	I_*i*105_(+)
Providing official residence of tourism such as hotels and inns	Hotel and motel per capita	I_*i*106_(+)
Providing tourism official residence with high quality	Three, four and five star hotels per capita	I_*i*107_(+)
Providing restaurants with high quality food and services	Restaurant per capita	I_*i*108_(+)
Information access	Information centers for tourists (per capita)	I_*i*109_(+)
Information access	Regional tourism websites	I_*i*110_(+)
Information access	The number of newspapers and local magazines	I_*i*111_(+)
Information access	Assessment tourism pace in national and regional region (0–10)	I_*i*112_(+)
Tourism seasonal demand	The ratio of low season tourists to high season	I_*i*113_(+)
Number of employed staff in tourism sector	Number of hotel employed staff	I_*i*114_(+)
Relative share of tourism employment to total employment in destination	Hotel employment to total employment	I_*i*115_(+)
Tourism employment	The percentage of employed women in tourism sector	I_*i*116_(+)
Tourism employment	Local employment people in tourism sector	I_*i*117_(+)
Generated employment in services sector	Service sector employed/total employment	I_*i*118_(+)
Transportation services	Transportation equipment per capita	(+) I_*i*119_
Airport access	Having or not having airport	I_*i*120_(+)
Highway access	Highway length/total area of region	I_*i*121_(+)
Road access	Road length/total area of region	(+)I_*i*122_
Railroad access	Having or not having railroad	I_*i*123_(+)
The occupation rate of residential places	Average of occupation rate	(+)I_*i*124_
Regional tourist attractions	The number of natural and historical attractions/region area	I_*i*125_(+)
Constructed roads in region	Total road length network/region area	I_*i*126_(+)
Protecting cultural heritage	Budget of renovation and restoration of cultural heritage (per capita)	I_*i*127_(+)
Access to required credit for tourism agencies	Evaluation of access to required credit for tourism agencies (0–10)	I_*i*128_(+)
The impact of tourism on booming regional crafts	Evaluation of local people's views from the impact of tourism on booming regional crafts (0–10)	I_*i*129_(+)

### Composite indicator of sustainable tourism

Composite indicator of sustainable tourism was calculated using the principal component analysis (PCA) defined as follows; N is the destination and J is the main factor that had indicator with *I*_*ij*_ value. At first step, indicators were presented and in the second step the value of each indicator was calculated (quantitative indicators by existing information and data and qualitative indicators using experts' opinions). In the third step, indicators were normalized using Eq. 1 [9, 16]:

1 $$ I{N}_{ij}=\frac{I_{ij}- min}{max- min} $$

It caused all values to be between 0 and 1 and did not affect the results. When the system became normal, finally Eq. 2 was used to make the composite indicator:

$$ D P{C}_i={\displaystyle \sum_{j=1}^q}\left[ V{E}_j\left({\displaystyle \sum_{i=1}^p} I{N}_{i j}\left| Cor{r}_{j i}\right|\right)\right] $$

i = 1,…, n, where n is the number of observations, p is the number of main indicators, q is the number factors and main selected component, VE_j_ is the explained variance by jth component, corr_ij_ is correlation between ith indicator and jth component. IN_ij_ is the value of normal indicator and finally calculating DPC_i_ (distance-principal component) that the value more than DPC_i_ shows the higher sustainability in each dimension. The composite indicator of sustainable tourism is easily interpretable so it did not need to understand the complex computing relationship.

Using made DPC indicator, the general sustainability indicator was analyzed in the next step by maximizing the target function for each destination by linear programming [16]:

3 $$ L P D P{C}_i= M a{x}_w{\displaystyle {\sum}_{j=1}^d{w}_j^i} D P{C}_{i j} $$

Subject to:

$$ \begin{array}{l}\begin{array}{cc}\hfill {\displaystyle \sum_{j=1}^d}{w}_j^i DP{C}_{i j}\le 1\hfill & \hfill\ i=1,\dots, n\hfill \end{array}\hfill \\ {}\begin{array}{ccc}\hfill {w}_j^i DP{C}_{i j}\ge {\omega}_i\hfill & \hfill\ i=1,\dots, n\hfill & \hfill j=1,\dots, d\hfill \end{array}\hfill \\ {}\begin{array}{cc}\hfill {w}_j^i\ge 0\hfill & \hfill j=1,\dots, d\hfill \end{array}\hfill \end{array} $$

Where *w*_*j*_^*i*^ are the weights for the observations i, d is the number of tourism dimension in this study: social, economic and environmental aspect environmental health, *DPC*_*ij*_ is the sustainable indicator in tourism dimension (calculated by Eq. 2. represents the jth dimension indicator for the ith observation) in tourism destination. ${\omega}_i$ is a real number (the minimum allowed weight provided by experts). This restriction caused all aspects (dimensions) of sustainable tourism to be considered. The advantage of this method was that the weights were determined by programming model and their views were not involved. *LPDPC*_*i*_ (Linear programming distance-principal component). The indicator value (*LPDPC*_*i*_) was between zero and one [16]. Number one represented the best and the most stable situation and zero represented the worst stable situation. *LPDPC*_*i*_ was calculated with the aim of maximizing the stability at each destination by using DPC_ij_ indicator, accordingly, destinations could be ranked and the highest and lowest sustainable places could be identified.

Gams, SPSS, Excel software and statistical methods were used to analyze data.

## Results

Cronbach’s alpha coefficient (stability) was calculated 0.9, so stability was acceptable and confirmed validity questionnaires.

**Fig. 2:**
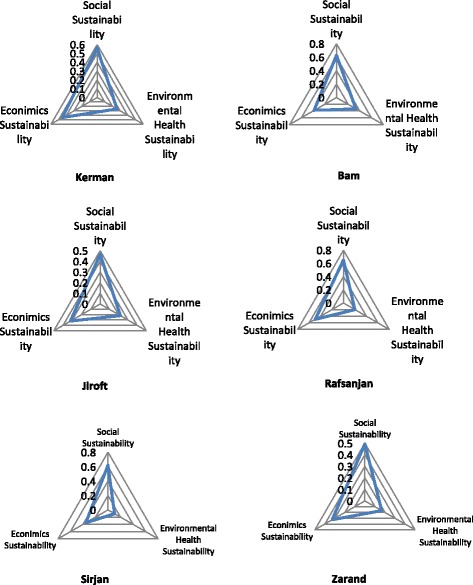
Sustainability dimensions of sustainable tourism

**Fig. 3:**
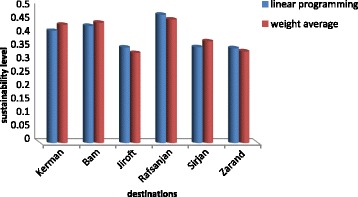
The sustainability of tourism destinations by weight average and linear programming method

Sustainable results in all tourism dimensions (environmental health, social and economic) were presented in Table 4 and Fig. 2; they showed all aspects of the low level of sustainability (0 was the worst situation and 1 was the best conditions) and environmental dimension (so environmental health) of sustainability was the most inappropriate situation. Using a linear programming model and experts’ survey, results of sustainability in tourism destinations (6 cities under study) were shown in Table 5. Moreover, Fig. 3; compared the sustainability of tourism destinations.

**Table 4:** Tourism dimensional (aspects) sustainability

City	Social DPC	Rank	Economic DPC	Rank	Environmental & Environmental health DPC	Rank
Kerman	0.56	4	0.45	2	0.25	2
Bam	0.62	2	0.38	3	0.33	1
Jiroft	0.46	6	0.32	5	0.21	3
Rafsanjan	0.64	1	0.49	1	0.19	4
Sirjan	0.61	3	0.35	4	0.10	6
Zarand	0.49	5	0.32	5	0.16	5

**Table 5:** Tourism sustainability destinations

City	Weight average (level sustainability)	Sustainability ranking	*LPDPC*_*i*_	Sustainability ranking
Kerman	0.4391	3	0.4166	3
Bam	0.4469	2	0.4347	2
Jiroft	0.3347	6	0.3546	5
Rafsanjan	0.4582	1	0.4761	1
Sirjan	0.3779	4	0.3588	4
Zarand	0.3402	5	0.3521	6

## Discussion

Tourists travel to tourist destinations or regions that have both regional attractions and life, financial and health security. Although tourists travel with different purposes, they consider public health a very important issue and prefer to travel to areas with safer environment. Tourist regions that have air and water pollution cannot retain the tourists’ health and therefore cannot attract tourists and will be far away from the sustainable development goals. Water pollution is a major issue because contaminated water disrupts the balance of the ecosystem and brings irreparable damages to it. The quality of drinking water has a significant impact on public health. One of the major concerns of tourists is water pollution because of its toxic effects on humans, animals and plants (and subsequently food contamination). Water pollution contaminates the agricultural lands, crops and livestock, produces unhealthy food and threatens the public health. Improving water quality and reducing the pollution is important for two reasons both of which are essential in terms of tourism: 1. human health (residents and tourists) and 2. Environmental protection. Air pollution endangers human and animal health and even vegetation, causes erosion and threatens historic monuments and cultural heritage. In recent decades, air pollution and smog have become an environmental problem especially in big cities; nowadays, tiny dust particles have also worsened the situation. Therefore, it is necessary to consider air pollution seriously so as to boost sustainable tourism industry. It is obvious that areas with high pollution will not be chosen for tourism. Since tourists use fossil fuel (concerning high consumption of such fuels in Iran transportation system) for their transportation, the environment will be severely damaged. Noise pollution is also an important factor in tourism because increased noise can increase stress and cause severe crisis for people. Therefore, this indicator is considered an important factor influencing tourism because cities with high noise pollution cannot become target destinations for tourists who seek recreation and leisure. Municipal wastes and effluents, sewage and wastes destroy the appearance of cities and tourist regions and are the cause of increased air and water pollution. Thus, when the environmental management (such as wastewater management, waste water and waste disposal) is inefficient, the possibility of attracting tourists is low. It is a mutual relationship between tourism and pollution; if there is high contamination in the area, it will endanger the health of tourists and residents and therefore tourists lose their intentions to travel to these regions. On the other hand, the mass presence of tourists (untrained and unmanaged) will increase the production of waste and increase air and water pollution. Therefore, it is necessary to consider a proper wastewater treatment system and a garbage collection system as indicators of sustainability. Soil erosion by water and wind reduces the soil fertility and soil structure degradation and permeability, creates ugly scenes, increases the likelihood of flooding and sediment production, harms the nature, the environment and the economy and reduces tourist attractions. It must be noted that most cities of Kerman have desert attractions in which erosion is completely obvious; this reduces tourism in these areas. The maintenance of biodiversity and health of plant and animal species lead to equitable distribution of biological resources between generations, and its sustainability can increase the livelihood opportunities of people from different ways like the ecotourism in a healthy environment. Tourism and biodiversity are interconnected; if we only look at it from the economical point of view, developing tourism without proper management is a threat to biodiversity of region; when we lose the health of species and biodiversity, the region will lose its capability of attracting tourists and their economic value creation. Also, preserving rare and endangered plant and animal species could attract ecotourism in a good way. Therefore, it is necessary to consider these factors in sustainable tourism. Different urban areas require the development of green space to be able to maintain their stability. Green spaces have ecological and environmental functions, partly deal with air pollution and improve their standard of living and tourism in which the density construction must also be taken into consideration to preserve the balance of nature. The green space per capita is one of the most important indicators of development of the societies in which the index of green space per capita must be considered in sustainability issue. The administrative environmental systems and improved management are essential to reduce the negative impacts of tourism activities. In general, vitality of ecosystem is a competitive factor attracting tourists in different tourism destinations.

The present study showed that unfortunately the opportunities for tourism were not used appropriately in the city and tourist destinations concerning sustainable development goals. Having multiple factors and tourist attractions, Kerman couldn’t attract many tourists, and these cities were not able to have a proper tourist attraction. Moreover, most indicators of these cities did not have appropriate levels (average sustainability 0.5). Therefore, these indicators were the tools to analyze the strengths and weaknesses of tourist attraction and the sustainable tourism levels in these cities. Thus, it could be concluded that the social dimension had the highest sustainability rank, while the environmental dimension had the lowest one; it showed low importance of the environmental dimension among these destinations. The results of this study were inconsistent (as expected) with the results of studies conducted by Blancas et al. [9], perez et al.[16] and Lozano-Oyola et al. [17] due to strong sustainable development, environmental protection and health issues in developed countries compared to the developing countries.

## Conclusion

Based on the studies conducted in this area, sustainable tourism has various environmental health, social – cultural and economic aspects. Therefore, to assess sustainable tourism in intended cities according to the sustainable development goals, it is necessary to consider an inclusive and comprehensive system of parameters and use this analysis deeply in the sustainable tourism. The results indicated that none of the intended cities had acceptable sustainable levels, and that they were far from it. Moreover, they revealed that the environmental aspect was not considered seriously and was much worse than other aspects. One of the essential factors was giving free environmental services and nature to human beings that led to inappropriate overuse, destruction and reduction of the levels of environmental health and environmental sustainability. Having found the ranking of cities by these indicators, the sustainable tourism of cities can improve using good policies and plans made by policy-makers and managers. If the environmental health aspect is taken into more account, the sustainability level will increase significantly. The relationship between tourism and the environmental health should be organized toward sustainable development so that we can have stable environment in the long term and we should not let tourism destroy natural resources and damage job creation in future. Since most data was quantitative in making composite indicators, the reliability and comparability of destinations would increase. Therefore, this method is one of the most appropriate methods which can be used to analyzed sustainable tourism, sustainable health environment and environment of destinations and can be a way to achieve sustainable development.

## Abbreviations

DPC: Distance- principal component
LPDPC: Linear programming distance-principal component
PCA: Principal component analysis
WTO: World tourism organization
